# Long-term adverse effects following COVID-19 vaccination: A comparative analysis of Oxford AstraZeneca, Pfizer-BioNTech, and Moderna vaccines

**DOI:** 10.1097/MD.0000000000050282

**Published:** 2026-08-28

**Authors:** Jehad A. Aldali, Sultan Ayoub Meo

**Affiliations:** a Department of Pathology, College of Medicine, Imam Mohammad Ibn Saud Islamic University (IMSIU), Riyadh, Saudi Arabia; b Department of Physiology, College of Medicine, King Saud University, Riyadh, Saudi Arabia.

**Keywords:** adverse symptoms, COVID-19 vaccination, Moderna, Oxford AstraZeneca, Pfizer-BioNTech

## Abstract

During the COVID-19 pandemic, vaccinations were highly beneficial to public health, preventing disease and reducing mortality. This study aimed to investigate long-term adverse clinical symptoms following COVID-19 vaccination with the Oxford AstraZeneca (ChAdOx1 nCoV-19, Oxford), Pfizer-BioNTech (BNT162b2), and Moderna (mRNA-1273) vaccines. In this cross-sectional study, the total number of participants was 429; among them, 243 (56.6%) were male, and 186 (43.4%) were female. An online Google Form was prepared in both Arabic and English and distributed to participants who received the Oxford AstraZeneca, Pfizer-BioNTech, and Moderna vaccines. The most common long-term adverse effects reported after COVID-19 vaccination were chronic fatigue, impaired cognitive function, and menstrual irregularity. The Moderna vaccine had a higher self-reported symptom rate among male participants (50–61.5%) than among female participants (33.3–28%). Chronic fatigue was more frequent among Oxford AstraZeneca recipients (34.7%) than among Moderna (22.2%) and Pfizer-BioNTech (27.7%) recipients. The second dose was associated with slightly higher rates of chronic fatigue among Oxford AstraZeneca recipients (38.9%) than among Moderna (25%) and Pfizer-BioNTech recipients (27.3%). For cognitive problems, the prevalence was similar across all brands (25%). Menstrual irregularity was slightly higher among females who received Pfizer-BioNTech (14.8%) than among Oxford AstraZeneca recipients (18.9%). The highest proportion of recipients who were infected after vaccination was among those who received the Oxford AstraZeneca vaccine (46.3%). In conclusion, the most commonly reported long-term adverse effects after COVID-19 vaccination were chronic fatigue, cognitive impairment, and menstrual irregularity. Further large-scale studies are required to provide more conclusive evidence.

## 1. Introduction

The severe acute respiratory syndrome coronavirus 2 (SARS-CoV-2) pandemic has caused widespread damage and posed challenges for countries worldwide.^[1,2]^ SARS-CoV-2 is a highly contagious virus that causes varying levels of infection and mortality in outbreak settings.^[1,2]^ Health experts and global lawmakers have adopted preventive measures to fight this pandemic. To prevent and eliminate the disease, measures included lockdowns, mask mandates, social distancing, and massive vaccination efforts. Throughout the COVID-19 pandemic, hospitals and communities implemented infection control measures to reduce the risk of virus transmission, and safe vaccines were developed.^[2,3]^ However, the vaccines recommended by the World Health Organization do not confer complete protection against the disease.^[3-5]^ Consequently, many countries recommend administering more than 1 dose.^[6]^ As vaccination efforts progressed globally, many countries, particularly the Kingdom of Saudi Arabia, were among the first to implement preventive measures, such as making vaccination against SARS-CoV-2 (COVID-19) mandatory for the first, second, and third doses.^[7,8]^ The Saudi Food and Drug Authority approved 2 different vaccine schedules for coronavirus disease (COVID-19). Pfizer-BioNTech developed and released an RNA-based vaccine on December 17, 2020, whereas Oxford-AstraZeneca developed and released a liposome nanoparticle-encapsulated vaccine encoding the SARS-CoV-2 spike protein in February 2021.^[9]^ The Moderna vaccine was licensed in Saudi Arabia on July 16, 2021.^[10]^ These SARS-CoV-2 vaccines substantially reduced mortality.^[10]^ The adverse effects associated with these COVID-19 vaccines include headache, pain at the injection site, inflammation and redness, and muscle and joint pain. These adverse effects ranged from mild to moderate in severity.^[11,12]^ The current state of knowledge regarding postvaccination sequelae in the general population is minimal. Such information is required to increase public confidence in the safety of COVID-19 vaccines, reduce concerns about different vaccine types, and support rapid vaccination initiatives to combat COVID-19. This study aimed to investigate the long-term adverse clinical symptoms following COVID-19 vaccination with the Oxford AstraZeneca (ChAdOx1 nCoV-19, Oxford), Pfizer-BioNTech (BNT162b2), and Moderna (mRNA-1273) vaccines. The long-term adverse effects were defined as self-reported symptoms that occurred after COVID-19 vaccination and persisted or recurred within 6 months.

## 2. Materials and methods

### 2.1. Study design and settings

This cross-sectional study was conducted at the Department of Pathology, College of Medicine, Imam Muhammad bin Saud Islamic University, Riyadh, Saudi Arabia, from September 10 to October 28, 2025.

### 2.2. Study participants

The study participants included people living in Saudi Arabia who received 3 doses of the COVID-19 vaccines authorized by the Saudi Ministry of Health. Participants were selected to represent various age groups, genders, and health backgrounds, thereby encompassing diverse experiences. The eligibility criteria required that participants be over 18 years of age, have received 3 doses of vaccination, and be willing to provide comprehensive information about their health after vaccination. The study sought to identify delayed postvaccination self-reported adverse effects, including neurological, cardiovascular, and autoimmune symptoms, and to evaluate their prevalence and duration.

### 2.3. Sample size

The sample size was calculated using the “Raosoft” power calculator. The Riyadh region in Saudi Arabia has a population of approximately 80,00,000, of whom 49% are female – a sample size of 385 was sufficient to achieve 95% confidence at a 5% margin of error. In total, 429 people participated in the study.

### 2.4. Inclusion and exclusion criteria

The inclusion and exclusion criteria ensured the study population was relevant and appropriate. Individuals aged 18 years or older who are residents of Saudi Arabia and have received 3 doses of a COVID-19 vaccine approved by the Saudi Ministry of Health, are willing to provide informed consent and participate in the study, and have the ability to complete questionnaires. However, participants who have not received any COVID-19 vaccine were excluded from the study. Participants who experienced severe adverse effects and required hospitalization immediately following vaccination were excluded. Those who were unable to provide informed consent or complete the required study procedure were excluded from the study.

### 2.5. Study questionnaire

In this cross-sectional study, an online survey, both in Arabic and English, was established and distributed via a Google Form. The survey was administered to participants who received the Moderna, Pfizer-BioNTech, and Oxford AstraZeneca vaccines. The survey was distributed throughout the Kingdom of Saudi Arabia. The online survey was designed and disseminated via social media platforms, such as WhatsApp, and emails to universities and public health staff. When accessing the online questionnaire, participants were asked to carefully review a comprehensive explanation of the study objectives before providing informed consent via a mandatory electronic consent form. This form contained the required information regarding voluntary participation and anonymity. To facilitate communication between the participants and researchers, an email address was created. Data collected from the online survey were stored securely in a confidential file after the survey was completed. The structured online study was divided into 2 sections: the first aimed to collect participants’ demographic details (gender, education, age, and nationality) and their SARS-CoV-2 infection status (including chronic conditions, prior COVID-19 infection, and medication use). The second section aimed to examine the type of vaccine administered, identify the late-reported adverse effects of COVID-19 vaccination (first, second, or third dose), and assess their duration. The survey excluded individuals who were not vaccinated with 3 doses or who received vaccines other than Pfizer-BioNTech, Oxford AstraZeneca, or Moderna. The sample size was determined using Raosoft software, which indicated that 385 participants were sufficient to achieve a 95% confidence level and a 5.35% margin of error.

### 2.6. Ethical approval and statistical analysis

The Institutional Review Board (IRB) at Imam Mohammad Ibn Saud Islamic University reviewed and approved this research project (Ref # 856/2025). The statistical analysis was performed using the Statistical Package for the Social Sciences (SPSS) Version 25 (IBM, Armonk). For descriptive statistics, significance was assessed for qualitative data with *P*-values ≤ .05. Comparing the adverse effects of different vaccine types, qualitative data from descriptive statistics revealed various late adverse effects associated with COVID-19 vaccination.

## 3. Results

### 3.1. Demographic characteristics of study participants

We present and analyze all possible adverse effects experienced by vaccinated individuals, providing a comprehensive perspective. In this study, 429 participants completed the survey, which was distributed among people of different ages, genders, nationalities, educational qualifications, and smoking statuses (Table 1). Among the participants, 43.4% were female, and 56.6% were male. Age categories were divided into 5 groups: 18 to 25, 26 to 30, 31 to 40, 41 to 50, and >60 years. The most prevalent age group was those aged 31 to 40 years, accounting for 28% of the study population. By nationality, 371 participants (86.5%) were Saudi, and the remaining 58 (13.5%) were non-Saudi. In response to an inquiry regarding smoking habits, 356 participants (83.0%) were identified as smokers, and 73 participants (17.0%) were identified as nonsmokers. The predominant educational qualification was a bachelor’s degree, accounting for 52.4%. The second most prevalent educational qualification was a master’s degree (20.7%), followed by a high school diploma (15.9%). Table 1 summarizes the participants’ general demographic characteristics.

**Table 1:** Demographic characteristics of study participants (n = 42).

Variables	Frequency	Per cent (%)
Gender		
Male	243	56.6
Female	186	43.4
Age (yr)		
18–25	114	26.6
26–30	52	12.1
31–40	120	28
41–50	65	15.2
50–60	50	11.7
>60	28	6.5
Nationality		
Saudi	371	86.5
Non-Saudi	58	13.5
Education qualification		
Bachelors	225	52.4
Masters	89	20.7
PhD	47	11
School	68	15.9
Smoking history		
Smokers	356	83.0
Nonsmokers	73	17.0
Overall health before receiving COVID-19 vaccines		
Excellent	311	72.5
Fair	19	4.4
Good	96	22.4
Poor	2	0.5

### 3.2. Adverse effects of COVID-19 vaccination according to age and gender

Among the study respondents who received the COVID-19 vaccine, 55.5% reported no change in their health, whereas 44.5% reported changes. Table 2 examines the reported adverse effects of COVID-19 vaccination on participants’ general well-being by gender (male or female). The Moderna vaccine had higher self-reported clinical symptom rates among male participants (50–61.5%) than among female participants (33.3–28%) for both the first and second doses, with *P*-values of 0.739 and 0.309, indicating that the difference was not statistically significant.

**Table 2:** The adverse effects of COVID-19 vaccines in males and females.

Female			Observed changes in overall health since receiving the COVID-19 vaccine			*P*-value
			No		Yes	
COVID-19 vaccination first dose	Moderna		66.7%		33.3%	.739
	Oxford AstraZeneca		51.4%		48.6%	
	Pfizer-BioNTech		47.3%		52.7%	
COVID-19 vaccination second dose	Moderna		71.4%		28.6%	.309
	Oxford AstraZeneca		54.1%		45.9%	
	Pfizer-BioNTech		45.8%		54.2%	
COVID-19 vaccination third dose	Moderna		52.4%		47.6%	.229
	Oxford AstraZeneca		61.5%		38.5%	
	Pfizer-BioNTech		64.7%		35.3%	

Male						*P*-value
COVID-19 vaccination first dose		Moderna		50.0%	50.0%	.768
		Oxford AstraZeneca		63.8%	36.2%	
		Pfizer-BioNTech		60.3%	39.7%	
COVID-19 vaccination second dosage		Moderna		38.5%	61.5%	.233
		Oxford AstraZeneca		62.9%	37.1%	
		Pfizer-BioNTech		62.1%	37.9%	
COVID-19 vaccination third dose		Moderna		61.9%	38.1%	.473
		Oxford AstraZeneca		48.5%	51.5%	
		Pfizer-BioNTech		64.3%	35.7%	

In contrast, the Oxford-AstraZeneca and Pfizer-BioNTech vaccines were associated with a higher reported incidence of adverse clinical symptoms among females. For the third dose, the Moderna vaccine had a greater impact on females (46.6%) than on males (38.1%), whereas the Pfizer-BioNTech vaccine affected both genders equally. In contrast, the Oxford-AstraZeneca vaccine had more substantial adverse effects in males than in females. There were no significant differences in health effects across vaccine brands or doses.

Table 3 presents the adverse effects of vaccination type on participants’ overall well-being by age. Upon administration of the first dose, the Moderna vaccine elicited more pronounced clinical symptoms among participants aged 31 to 40 years (75%) and those aged 60 years and above (25%). The first Oxford AstraZeneca vaccine dose was administered to study participants aged 26 to 31 years (15.4%) and 41 to 50 years (20.5%). The first Pfizer-BioNTech vaccination was administered to participants aged 18 to 60 years. The *P*-value of .001 indicates a statistically significant difference in the age distribution of first-dose recipients across the vaccine brands. The second Moderna vaccine dose was administered to participants aged 31 to 40, 45 to 50, and >60 years. The second Oxford-AstraZeneca vaccine dose had a more pronounced effect among participants aged 26 to 40 years. The second Pfizer-BioNTech vaccine dose was administered to participants aged 18 to 25, 31 to 40, and 50 to 60 years. The *P*-value of .088 indicates no statistically significant difference in age distribution among vaccine types for the second dose. The third Moderna vaccine dose was administered to participants aged 31 to 40 and 50 to 60 years. In contrast, the third Oxford AstraZeneca vaccine dose was administered to participants aged 31 to 40, 18 to 25, and 41 to 50. The third Pfizer-BioNTech vaccine was administered to participants aged 18 to 25, 31 to 40, and 41 to 50 years. The age distribution of third-dose vaccine brands is not statistically significant (*P*-value = .866).

**Table 3:** Adverse effects of COVID-19 vaccine on overall health according to age.

Vaccination		Age						*P*-value
		18–25	26–30	31–40	41–50	51–60	>60	
COVID-19 vaccination first dose	Moderna	0.0%	0.0%	75.0%	0.0%	0.0%	25.0%	.001
	Oxford AstraZeneca	0.0%	15.4%	41.0%	20.5%	7.7%	7.7%	
	Pfizer-BioNTech	7.70%	10.8%	24.3%	14.9%	16.9%	5.4%	
COVID-19 vaccination second dose	Moderna	27.70%	10.0%	30.0%	20.0%	10.0%	20.0%	.088
	Oxford AstraZeneca	10.0%	20.0%	40.0%	10.0%	13.3%	6.7%	
	Pfizer-BioNTech	28.5%	9.9%	26.5%	16.6%	15.2%	5.3%	
COVID-19 vaccination third dose	Moderna	9.1%	13.6%	45.5%	4.5%	22.7%	4.5%	.866
	Oxford AstraZeneca	18.80%	12.5%	31.3%	18.8%	12.5%	6.3%	
	Pfizer-BioNTech	20.5%	11.1%	28.2%	18.8%	14.5%	6.8%	

### 3.3. Most common adverse effects of COVID-19 vaccination

The most common adverse reactions are presented in Table 4, organized by the types of vaccines administered at each dose. Notably, most recipients reported no allergic reactions across all vaccine brands, with Moderna showing the highest proportion of “No” responses (88.9%) compared with Pfizer-BioNTech (79.4%) and Oxford-AstraZeneca (75.8%). Chronic fatigue was reported more frequently among Oxford-AstraZeneca recipients (34.7%) than among Moderna (22.2%) and Pfizer-BioNTech (27.7%) recipients. For cognitive problems, the prevalence was similar across all brands, with approximately 25% of the recipients reporting cognitive issues. None of the conditions showed statistically significant differences among the vaccine brands for the first dose; *P*-values exceeded .05.

**Table 4:** Most frequent adverse reactions of COVID-19 vaccines.

Clinical Symptoms	First dose of COVID-19 vaccination				*P*-value	Second dose of COVID-19 vaccination			*P*-value	Third dose of COVID-19 vaccination			*P*-value
	Moderna		Oxford AstraZeneca	Pfizer-BioNTech		Moderna	Oxford AstraZeneca	Pfizer-BioNTech		Moderna	Oxford AstraZeneca	Pfizer-BioNTech	
Allergic reactions	No	88.9%	75.8%	79.4%	.568	75.0%	76.4%	79.5%	.767	71.7%	80.0%	79.9%	.658
	Yes	11.1%	24.2%	20.6%		25.0%	23.6%	20.5%		28.3%	20.0%	20.1%	
Chronic fatigue	No	77.8%	65.3%	72.3%	.372	75.0%	61.1%	72.7%	.133	78.3%	71.1%	70.1%	.698
	Yes	22.2%	34.7%	27.7%		25.0%	38.9%	27.3%		21.7%	28.9%	29.9%	
Cognitive/ memory problems	No	77.8%	73.7%	74.2%	.964	60.0%	76.4%	74.5%	.317	71.7%	82.2%	74.0%	.573
	Yes	22.2%	26.3%	25.8%		40.0%	23.6%	25.5%		28.3%	17.8%	26.0%	
Hypertension or Hypotension	No	100.0%	86.3%	88.3%	.468	95.0%	83.3%	88.7%	.273	87.0%	91.1%	87.4%	.878
	Yes	0.0%	13.7%	11.7%		5.0%	16.7%	11.3%		13.0%	8.9%	12.6%	
Heart disorders	No	100.0%	88.4%	92.9%	.243	95.0%	94.4%	91.4%	.606	89.1%	84.4%	92.5%	.094
	Yes	0.0%	11.6%	7.1%		5.0%	5.6%	8.6%		10.9%	15.6%	7.5%	
Autoimmune disorders	No	100.0%	96.8%	92.9%	.276	95.0%	94.4%	93.8%	.956	91.3%	95.6%	94.1%	.854
	Yes	0.0%	3.2%	7.1%		5.0%	5.6%	6.2%		8.7%	4.4%	5.9%	
Respiratory disorders	No	100.0%	80.0%	85.8%	.166	95.0%	84.7%	84.3%	.429	84.8%	84.4%	85.0%	.999
	Yes	0.0%	20.0%	14.2%		5.0%	15.3%	15.7%		15.2%	15.6%	15.0%	
Any change on the skin	No	100.0%	87.4%	88.9%	.512	100.0%	88.9%	88.1%	.262	82.6%	84.4%	90.9%	.278
	Yes	0.0%	12.6%	11.1%		0.0%	11.1%	11.9%		17.4%	15.6%	9.1%	

The second dose had slightly higher rates of chronic fatigue among Oxford-AstraZeneca recipients (38.9%) than among Moderna (25%) and Pfizer-BioNTech (27.3%) recipients, although the difference was not statistically significant (*P* = .133). Allergic reactions and cognitive problems were evenly distributed across all brands. For the third dose, Oxford-AstraZeneca recipients had the lowest prevalence of allergic reactions (20%), whereas respiratory and skin issues were evenly distributed across all brands. No statistically significant differences in any health condition were observed across vaccine types, as all *P*-values were >.05.

### 3.4. Adverse effects of COVID-19 vaccine on female menstrual cycles

Table 5 presents the relationship between irregular menstrual cycles and the number of COVID-19 vaccination doses. For the first dose, menstrual irregularity was reported by 0.0% of female Moderna recipients, 43.9% of female Oxford AstraZeneca recipients, and 31.6% of female Pfizer-BioNTech recipients. There was no statistically significant difference between vaccine groups for the first dose (*P* = .382).

**Table 5:** Menstrual irregularity after COVID-19 vaccination among female participants.

COVID-19 vaccine dose	Vaccine type	No menstrual irregularity among females	Menstrual irregularity among females	*P*-value
First dose	Moderna	100.0%	0.0%	.382
First dose	Oxford AstraZeneca	56.1%	43.9%	
First dose	Pfizer-BioNTech	68.4%	31.6%	
Second dose	Moderna	87.5%	12.5%	.593
Second dose	Oxford AstraZeneca	64.8%	35.2%	
Second dose	Pfizer-BioNTech	65.6%	34.4%	
Third dose	Moderna	81.3%	18.7%	.012
Third dose	Oxford AstraZeneca	68.7%	31.3%	
Third dose	Pfizer-BioNTech	58.2%	41.8%	

For the second dose, menstrual irregularity was reported by 12.5% of female Moderna recipients, 35.2% of female Oxford AstraZeneca recipients, and 34.4% of female Pfizer-BioNTech recipients. The *P*-value was .593, also indicating no statistically significant difference between vaccine groups.

For the third dose, menstrual irregularity was reported by 18.7% of female Moderna recipients, 31.3% of female Oxford AstraZeneca recipients, and 41.8% of female Pfizer-BioNTech recipients. The *P*-value was .012, which indicates a statistically significant difference between vaccine groups for the third dose.

### 3.5. COVID-19 infection after COVID-19 vaccination

Table 6 presents the correlation between the administration of COVID-19 vaccines (Moderna, Oxford AstraZeneca, and Pfizer-BioNTech) and subsequent COVID-19 infection after each dose. The highest percentage of recipients who remained uninfected after the first dose was among Moderna recipients (77.8%), followed by Pfizer-BioNTech (58.2%) and Oxford AstraZeneca (53.7%). In contrast, the highest proportion of recipients who were infected after vaccination was among those who received the Oxford AstraZeneca vaccine (46.3%). There was no statistically significant correlation between the vaccine type administered and the infection following the first dose (*P*-value .343).

**Table 6:** Infection with COVID-19 following COVID-19 vaccination.

		After receiving the COVID-19 vaccination, 1 can still become reinfected with the virus.		*P*-value
		No	Yes	
COVID-19 vaccination: have you received the first dose?	Moderna	77.8%	22.2%	.343
	Oxford AstraZeneca	53.7%	46.3%	
	Pfizer-BioNTech	58.2%	41.8%	
COVID-19 vaccination, have you received the second dose?	Moderna	55.0%	45.0%	.962
	Oxford AstraZeneca	56.9%	43.1%	
	Pfizer-BioNTech	57.9%	42.1%	
COVID-19 vaccination, have you received the third dose?	Moderna	43.5%	56.5%	.144
	Oxford AstraZeneca	66.7%	33.3%	
	Pfizer-BioNTech	57.9%	42.1%	

For Oxford AstraZeneca and Pfizer-BioNTech, 43.1% and 42.1% of recipients, respectively, were infected after vaccination. The infection rates did not differ significantly after the second dose (*P*-value = .962). After the third dose, the Moderna trend reversed: 56.5% of recipients were infected, compared with 43.5% who were not. Oxford AstraZeneca (33.3%) and Pfizer-BioNTech (42.1%) exhibited lower infection rates. The third-dose *P*-value (0.144) suggests that these differences are not statistically significant. No significant correlation was found between COVID-19 vaccine type and postvaccination infection across all doses.

## 4. Discussion

The vaccines are highly beneficial to public health because they reduce mortality by preventing and controlling the transmission of infectious diseases.^[13]^ During the COVID-19 pandemic, many countries implemented strict vaccination policies across the public and private sectors, requiring travelers to be vaccinated before entering.^[14]^ Saudi Arabia promptly launched early vaccination efforts after the Saudi Ministry of Health and the World Health Organization (WHO) approved COVID-19 vaccines.^[2]^ Three vaccines licensed and launched in Saudi Arabia were Pfizer-BioNTech’s mRNA vaccine, Oxford-AstraZeneca’s vaccine, and Moderna’s vaccine, approved in May 2021.^[14]^

While the COVID-19 vaccine was launched, the public had both positive and negative concerns. Many believed it would protect lives and reduce the spread of the virus, while others were worried about adverse effects. Adverse events from COVID-19 vaccines cause concern and apprehension among the public. To maintain public trust in vaccination programs, healthcare professionals should monitor and address the adverse events.^[15]^ The literature has covered acute side effects of vaccines^[16]^; however, data on long-term adverse effects (LTAEs) have been scarce.

In this study, we evaluated the long-term adverse effects of COVID-19 vaccines in Saudi Arabia. The commonly reported long-term adverse effects after COVID-19 vaccination were chronic fatigue, cognitive impairment, and menstrual irregularity. The Moderna vaccine was associated with more clinical symptoms in male participants, whereas the Oxford-AstraZeneca and Pfizer-BioNTech vaccines were associated with more symptoms in female participants. Oxford-AstraZeneca recipients were more likely to experience chronic fatigue, particularly after the second dose. Female recipients of Pfizer-BioNTech had higher rates of menstrual irregularity than those receiving Oxford-AstraZeneca. Oxford-AstraZeneca recipients were more likely to experience postvaccination COVID-19 infection, whereas Moderna recipients had the highest proportion remaining uninfected after the first dose.

Dar-Odeh et al^[17]^ investigated long-term adverse events associated with the Pfizer-BioNTech, Sinopharm, and AstraZeneca COVID-19 vaccines. The study participants reported LTAEs, including fatigue, myalgia, arthralgia, headache, and menstrual disturbances. These study findings are moderately similar to our study findings.

In our study, for the first and second doses, most female participants reported no irregular menstrual cycles across all vaccine brands. Moderna consistently had the highest proportion of participants without issues, followed by Oxford-AstraZeneca and Pfizer-BioNTech. A small percentage of the female participants experienced menstrual irregularities, with slightly higher rates reported among the Pfizer-BioNTech recipients than among the recipients of the other vaccines. However, the differences between vaccine brands were not statistically significant, indicating that irregular menstrual cycles were evenly distributed across vaccine types.

For the third dose, differences between the vaccine brands became more apparent. Moderna and Oxford-AstraZeneca vaccine recipients were more likely to report no irregular menstrual cycles than Pfizer-BioNTech vaccine recipients. Conversely, a higher proportion of females who received the Pfizer-BioNTech vaccine experienced irregularities than those who received the other 2 vaccines. The statistically significant result suggests that the likelihood of irregular menstrual cycles may vary by vaccine brand for the third dose.^[18]^ In addition, screening results reveal various general health changes experienced by a sizable proportion of vaccinated individuals. The prevalence of reported adverse effects includes allergies. This result is similar to previous findings. For example, Kim et al^[19]^ found that the vaccine’s active antigen could cause topical reactions. However, allergic reactions to vaccines can also be caused by non-human proteins in the vaccine or by preservatives and stabilizers in the formulation. This study finds that commonly reported adverse clinical symptoms after vaccination were chronic fatigue and cognitive and memory impairment. The results of this study highlight the importance of providing comprehensive health care to individuals who experience postvaccination symptoms.

### 4.1. Study limitations

Despite its strengths, this study has certain limitations. The reliance on self-reported data is a significant limitation because it may introduce bias or inaccuracies stemming from participants’ subjective symptom interpretations and recall biases. This can influence the timing and accuracy of the reported adverse effects. In addition, although this study involves a substantial number of participants, it may not adequately capture the full range of rare or long-term effects, as certain issues may manifest over extended periods or in a smaller subset of the population. The ability to determine the actual long-term adverse effects of vaccines is also limited by the relatively short follow-up periods for specific reported adverse effects. Because the data were collected using a self-reported, cross-sectional online questionnaire, the study could not clinically confirm whether the reported symptoms were new onset after vaccination, persistent, recurrent, or related to preexisting conditions. We did not perform multivariable regression analyses in this manuscript, so the associations should be interpreted as unadjusted rather than independent effects. Therefore, the findings should be interpreted as self-reported symptoms after vaccination rather than as confirmed vaccine-related adverse effects.

## 5. Conclusion

The most commonly reported long-term adverse clinical symptoms after COVID-19 vaccination were chronic fatigue, cognitive impairment, and menstrual irregularity. The Moderna vaccine was associated with more clinical symptoms in male participants, whereas the Oxford-AstraZeneca and Pfizer-BioNTech vaccines were associated with more symptoms in female participants. Oxford-AstraZeneca recipients were more likely to experience chronic fatigue, particularly after the second dose. Female recipients of Pfizer-BioNTech had higher rates of menstrual irregularity than those receiving Oxford-AstraZeneca. Oxford-AstraZeneca recipients were more likely to experience postvaccination COVID-19 infection. Further large-scale studies are required to provide more conclusive evidence.

## Author contributions

**Conceptualization:** Jehad A. Aldali, Sultan Ayoub Meo.

**Data curation:** Jehad A. Aldali, Sultan Ayoub Meo.

**Formal analysis:** Jehad A. Aldali, Sultan Ayoub Meo.

**Funding acquisition:** Jehad A. Aldali.

**Investigation:** Jehad A. Aldali.

**Project administration:** Jehad A. Aldali.

**Supervision:** Jehad A. Aldali.

**Validation:** Jehad A. Aldali, Sultan Ayoub Meo.

**Writing – original draft:** Jehad A. Aldali, Sultan Ayoub Meo.

**Writing – review & editing:** Jehad A. Aldali, Sultan Ayoub Meo.
